# Coordinated Regulation of Orchard Variable-Rate Spraying: From Canopy Perception to Actuation—A Review

**DOI:** 10.3390/s26206431

**Published:** 2026-10-12

**Authors:** Shiqun Dai, Dengjun Tu, Xurui Fan, Ming Wang, Zhiyang Zhang, Wenrui Zhu, Li Jiang, Jianfei Zhou, Weidong Jia

**Affiliations:** 1 School of Agricultural Engineering, Jiangsu University, Zhenjiang 212013, China; daishiqun@ujs.edu.cn (S.D.); tudengjun@stmail.ujs.edu.cn (D.T.); fanxurui@stmail.ujs.edu.cn (X.F.); wangming@ujs.edu.cn (M.W.); 3232501014@stmail.ujs.edu.cn (Z.Z.); 3242203051@ujs.edu.cn (W.Z.); jiangliujs@ujs.edu.cn (L.J.); jfzhou@ujs.edu.cn (J.Z.); 2 Key Laboratory of Plant Protection Engineering, Ministry of Agriculture and Rural Affairs, Jiangsu University, Zhenjiang 212013, China

**Keywords:** precision pesticide application, LiDAR, machine vision, multispectral sensing, orchard mapping, spatial registration, prescription map, sensor–actuator mapping

## Abstract

Orchard variable-rate spraying increasingly depends on the coordinated conversion of heterogeneous perception outputs into spatially and temporally matched actuator commands, yet the evidence linking sensing improvements to measurable spray-performance gains remains fragmented. Rather than reviewing canopy perception technologies in isolation, this review focuses on the complete perception-to-actuation chain for coordinated orchard spraying and identifies which perception outputs are sufficiently actionable for regulation of liquid flow, airflow, nozzle timing, and spray trajectory. A structured narrative review of the peer-reviewed literature published up to August 2026 was organized by perception object, sensing modality, platform type, orchard system, and control relevance; representative studies were retained when they reported a clear linkage between sensed information and spraying decisions or provided transferable evidence for that linkage. Four perception-information classes are distinguished: canopy geometric profile, internal canopy structure, crop/plant-protection status, and spatial-geographic/operational context. LiDAR, RGB/RGB-D and binocular vision, multispectral/hyperspectral sensing, positioning, and multimodal fusion are compared according to the information they provide and their suitability for real-time control. Cross-study synthesis shows that the strongest evidence currently concerns canopy-volume- or density-based liquid-flow-rate regulation, while robust mappings for airflow demand, disease-severity-based dosage, and cross-season transfer remain less mature. Reported benefits include substantial pesticide savings in selected field or prototype studies, but the evidence is heterogeneous and often species-, platform-, and scenario-specific. The central research need is, therefore, not higher perception accuracy alone, but physically interpretable, latency-aware, spatially registered mappings from heterogeneous perception outputs to executable nozzle, liquid-flow, airflow, and trajectory commands. Concrete priorities include standardized structural descriptors, end-to-end delay compensation, transferable dose models, and benchmark field protocols for closed-loop validation.

## 1. Introduction

Spray application remains a principal means of pest and disease management in orchards, but its effectiveness is strongly constrained by the structural complexity and spatial heterogeneity of perennial tree canopies. Compared with relatively homogeneous field crops, orchard canopies exhibit substantial variability in tree size, three-dimensional architecture, foliage density, porosity, and target distribution. Air-assisted spraying can further modify canopy aerodynamics through leaf reorientation and deformation, thereby influencing droplet transport and deposition [1,2]. Consequently, fixed application rates, constant pressure, and uniform airflow are poorly suited to simultaneously achieving adequate canopy coverage, internal penetration, high pesticide-use efficiency, and limited off-target losses. Orchard and vineyard deposition studies likewise show that liquid flow rate, droplet spectrum, application technique, and ambient-wind-driven droplet transport jointly determine on-target deposition and off-target losses [3,4].

The development of precision agriculture has, therefore, accelerated the transition from uniform spraying toward target-adaptive and demand-oriented application. Representative laser-, vision-, and sensor-guided systems demonstrate that canopy presence, dimensions, density, and spatial position can be translated into nozzle switching or variable-flow commands [5,6]. At broader spatial scales, UAV-derived canopy-vigor information and three-dimensional reconstruction have been used to generate prescription maps for spatially variable pesticide application [7,8]. These developments indicate that variable-rate spraying is not merely a matter of regulating liquid flow rate; rather, it requires coordinated matching among target characteristics, spray dosage, airflow supply, and spatial actuation. Earlier canopy-adaptive systems established the engineering basis for this transition by linking tree occurrence, canopy distance, or canopy dimensions to automatic spray control [9,10], while canopy-based dose models demonstrated that orchard geometry can be used to adjust application volume [11,12].

Recent advances in sensing technologies have substantially expanded the information available for such control. UAV photogrammetry and image segmentation support orchard-scale estimation of individual-tree geometry [13], whereas LiDAR provides detailed three-dimensional information on canopy geometry and sectional density and can be combined with inertial and positioning measurements to improve target registration under uneven terrain [14,15]. RGB-D and binocular vision integrate semantic and geometric information for near-field canopy reconstruction and localization [16,17]. Beyond structural sensing, image and spectral methods increasingly support disease and physiological-status assessment [18,19]. Collectively, these technologies have moved orchard spraying from simple target-presence detection toward multidimensional characterization of canopy geometry, internal structure, crop status, and spatial context.

A recent *Sensors* review by Wang et al. systematically summarized orchard canopy perception technologies, dynamic canopy modeling, and their roles in variable-rate spraying [20]. Building on that perception-oriented foundation, the present review does not repeat a sensor-centered survey; instead, it treats canopy perception as the front end of a broader coordinated-regulation problem and concentrates on how heterogeneous outputs are converted into executable liquid-flow, airflow, nozzle-timing, trajectory, and prescription commands. Section 2 and Section 3 therefore retain only the control-oriented perception information and sensing methods needed to define regulation inputs, whereas Section 4 and the cross-study synthesis emphasize perception-to-actuation mapping, spatiotemporal coordination, and closed-loop validation.

However, improved perception does not necessarily translate directly into improved spraying performance. The practical value of sensing information depends on whether it can be converted reliably and rapidly into executable actuator commands. Recent studies have begun to establish such perception-to-actuation relationships by mapping canopy volume to PWM-controlled liquid flow rate [21], disease severity to variable-rate spraying decisions [22,23], and canopy density or structural resistance to airflow regulation [24,25,26,27]. More integrated approaches have further transformed three-dimensional canopy information into multidimensional prescription maps or jointly adjusted spray dosage, airflow, and spray direction according to target structure [28,29]. Nevertheless, most current mappings remain dependent on empirical coefficients, discrete classification thresholds, crop-specific calibration, or simplified mechanistic relationships. Their robustness is further challenged by illumination variability; occlusion; uneven terrain; canopy motion; environmental wind; sensing–actuation delays; and differences among cultivars, training systems, growth stages, and management practices. Moreover, sensing, prescription generation, and actuator control often operate at different spatial and temporal resolutions, creating additional difficulties in establishing a stable closed-loop relationship between canopy information and spray delivery. These limitations suggest that perception, decision making, and actuation should be considered as a coupled process rather than as independent technical modules.

To make the evidence base and table construction transparent, this review adopts a structured narrative-search and screening framework. Literature published up to August 2026 was identified through topic searches in Web of Science Core Collection, Scopus, and Google Scholar using combinations of “orchard”, “canopy”, “LiDAR”, “machine vision”, “RGB-D”, “multispectral”, “prescription map”, “variable-rate spraying”, “air-assisted spraying”, “PWM”, “positioning”, and “spatial registration” supplemented by backward and forward reference tracing. Peer-reviewed journal articles were prioritized. Studies were included when they (i) addressed orchard, vineyard, or closely comparable perennial-canopy systems; (ii) quantified perception information relevant to spray decisions, spatial registration, or actuator control; or (iii) provided mechanistic evidence directly transferable to perception-to-actuation mapping. Field-crop or non-spraying studies were retained only when they supplied a specific transferable sensing or mechanistic method and are explicitly identified as such. The review is, therefore, a structured narrative synthesis rather than a meta-analysis, because platforms, canopy systems, test conditions, and reported performance metrics are too heterogeneous for statistically valid pooling. Representative studies are synthesized by sensing and regulation pathways, while quantitative outcomes and evidence strength are compared where sufficient data are available.

To avoid ambiguity, terminology is standardized throughout this review. Variable-rate spraying denotes the overall control strategy; liquid flow rate denotes instantaneous liquid delivery (e.g., L min^−1^); application rate denotes area-normalized pesticide delivery (e.g., L ha^−1^); and spray volume denotes the total liquid consumed in a test or operation. Air velocity denotes local air speed, whereas air volume flow rate denotes volumetric airflow. Sprayer refers to the complete spraying machine, platform to its mobile carrier, and actuator to controllable components such as nozzles, valves, fans, and orientable spray mechanisms. Forward speed is used consistently for translational travel speed.

Accordingly, this review places coordinated regulation at the center of the analysis and treats orchard canopy and operational perception as the information basis for control. Perception information is classified into four control-oriented groups: geometric profile, internal canopy structure, status/prescription information, and spatial-geographic/operational context. Representative sensing technologies are evaluated in terms of information-acquisition capability, spatial scale, latency, environmental sensitivity, and control relevance. The review then examines how perception outputs are translated into nozzle switching, liquid-flow-rate allocation, spray-zone or trajectory adjustment, airflow-assisted regulation, and prescription-map-driven coordinated control. Finally, cross-study evidence is graded according to test conditions and quantitative outcomes, and concrete research questions are derived for perception-to-actuation mapping, delay compensation, transferable dose models, and standardized validation. This organization distinguishes the present review from perception-centered surveys by using sensing information primarily to explain how coordinated regulation is formulated, executed, and validated.

## 2. Control-Oriented Perception Information for Coordinated Orchard Spraying

This section retains only the perception objects and information classes that directly serve coordinated spray regulation. In addition to canopy geometry, internal structure, and crop status, spatial-geographic and operational-context perception is included because tree localization, platform–tree registration, motion state, and local environment determine where and when an actuator command is executed. The categories are control-oriented rather than sensor-oriented: the same sensing modality may contribute to several information classes.

### 2.1. Canopy Geometric Profile Information

Canopy geometric profile information represents one of the most direct perception outputs for orchard spray control. It includes tree height, canopy width and thickness, canopy volume, external boundary morphology, and relative target position [14,30]. These descriptors define the geometric extent of the target spraying area and support spatially matched nozzle control, spray-zone partitioning, and dose allocation. Visual and point-cloud reconstruction studies, including methods developed outside orchard spraying, are useful only insofar as they provide transferable geometry-recovery procedures [31,32]. Earlier ultrasonic and laser studies established practical links between canopy width or volume measurement and electronic application control in perennial crops [33,34]. Table 1 therefore selects representative studies spanning UAV-scale geometry mapping, ground-based semantic segmentation, and target-zone extraction rather than attempting to inventory every canopy-measurement paper.

**Figure 1:**
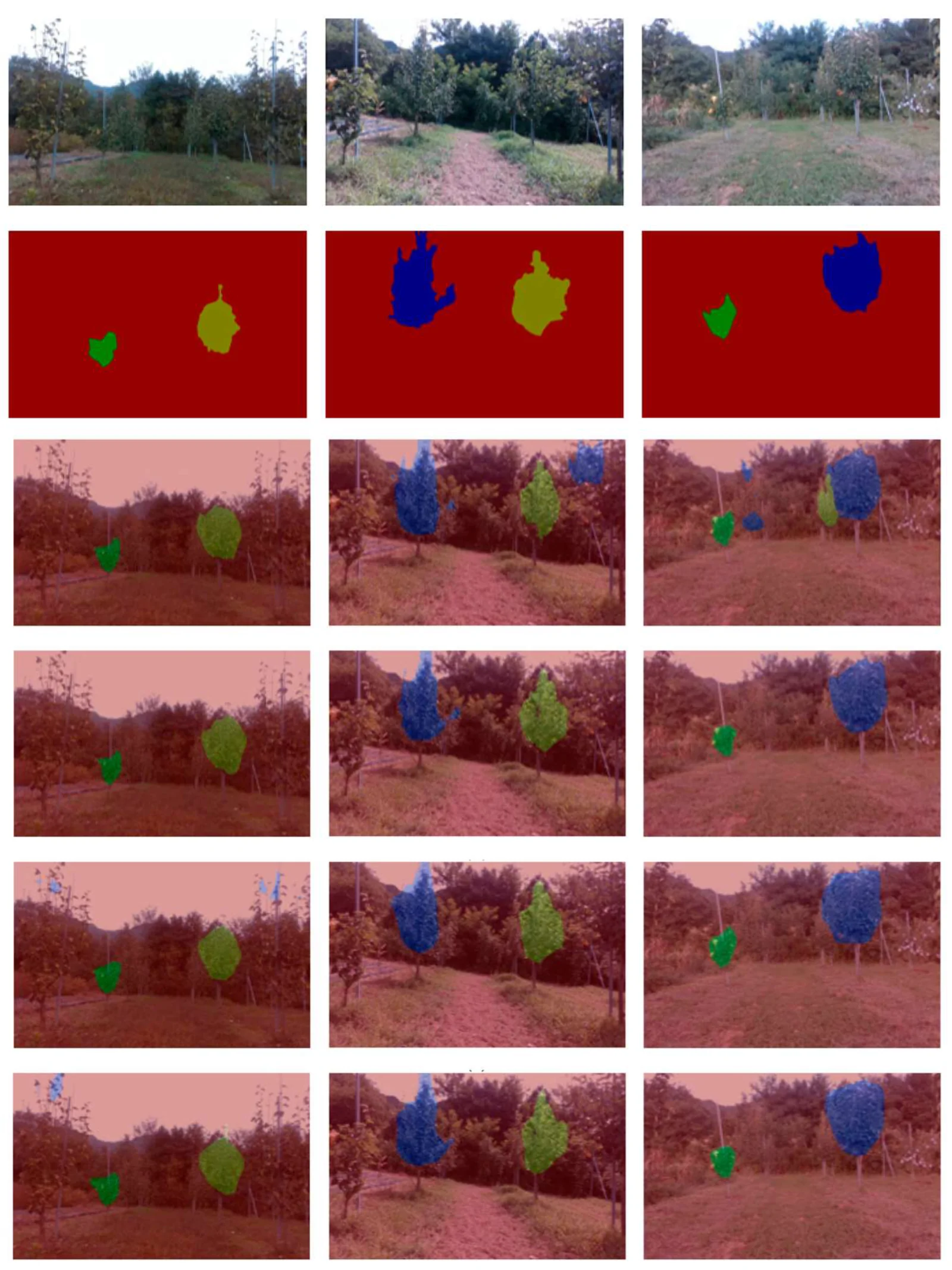
Semantic segmentation of apple tree canopy-size classes for automated variable-rate spraying. The panels compare raw images, ground-truth annotations, and predictions from four CNN-based segmentation configurations across paired small, medium, and large canopy classes. (Reprinted from Ref. [36] under the Creative Commons Attribution 4.0 International license (CC BY 4.0)).

With the deepening of orchard perception research, the role of geometric profile information has gradually expanded from spatial recognition of target presence to front-end input for spray dosage regulation. Gao et al. developed a real-time grapevine canopy volume perception system based on RGB-D vision and GPU-accelerated processing and used the estimated canopy volume to dynamically regulate PWM-driven spray flow rate. This enabled real-time matching between spray dosage and canopy spatial structure, indicating that canopy volume has evolved from a purely structural descriptor into an important basis for liquid-flow-rate allocation [21]. Zhang et al. further integrated fruit tree canopy volume information with disease detection results and developed a variable-rate spraying method based on the coordinated decision making of disease severity and canopy volume. This method was applied to variable-speed spray control for pear disease management, suggesting the potential to combine geometric quantification information with higher-level crop health status information as joint inputs for spray regulation [23]. Jin et al. further demonstrated CNN-based semantic segmentation for distinguishing small, medium, and large apple tree canopies in automated variable-rate spraying [36]; representative segmentation outputs are shown in Figure 1. In Figure 1, the three columns correspond to small, medium, and large canopy classes, respectively; from top to bottom, the rows show the original orchard images, ground-truth segmentation masks, and predictions generated by four CNN-based segmentation configurations. These studies indicate that, although geometric profile information is located at the basic layer of the perception chain, its role in control systems is extending from static recognition toward dynamic decision support. Terrestrial laser scanning has also been used to reconstruct orchard three-dimensional architecture and quantify crown structure, while early laser instruments and LiDAR-based deposition models demonstrated the control value of geometric descriptors [38,39,40,41].

However, geometric profile information alone cannot fully support high-precision spraying. External canopy boundaries define canopy extent but do not capture internal features such as branch–leaf distribution, local density, and resistance to droplet penetration. Moreover, boundary fuzziness and differences in sampling and reconstruction may reduce nozzle-switching stability. Thus, geometric information mainly supports spatial matching, whereas finer spray-rate and airflow regulation requires complementary structural, status, and prescription information.

### 2.2. Internal Canopy Structural Information

Compared with external canopy profiles, internal canopy structural information is more directly associated with the mechanisms of droplet deposition. It mainly includes foliage distribution, branch and leaf occlusion, local density, leaf area index, spatial porosity, and permeability at different canopy layers. Such information directly affects droplet transport pathways, penetration efficiency, and deposition outcomes within the canopy, and therefore, has greater value for variable liquid-flow-rate regulation and air-assisted control. From the perspective of spray mechanisms, whether droplets can effectively enter the canopy interior and form sufficient deposition at target layers depends not only on whether the external canopy region is covered, but also on whether the internal spatial structure facilitates droplet transport. Field and artificial-canopy experiments further demonstrate that tree architecture, spray quality, application rate, and sprayer configuration interact strongly with deposition profiles and canopy penetration [42,43,44,45].

**Figure 2:**
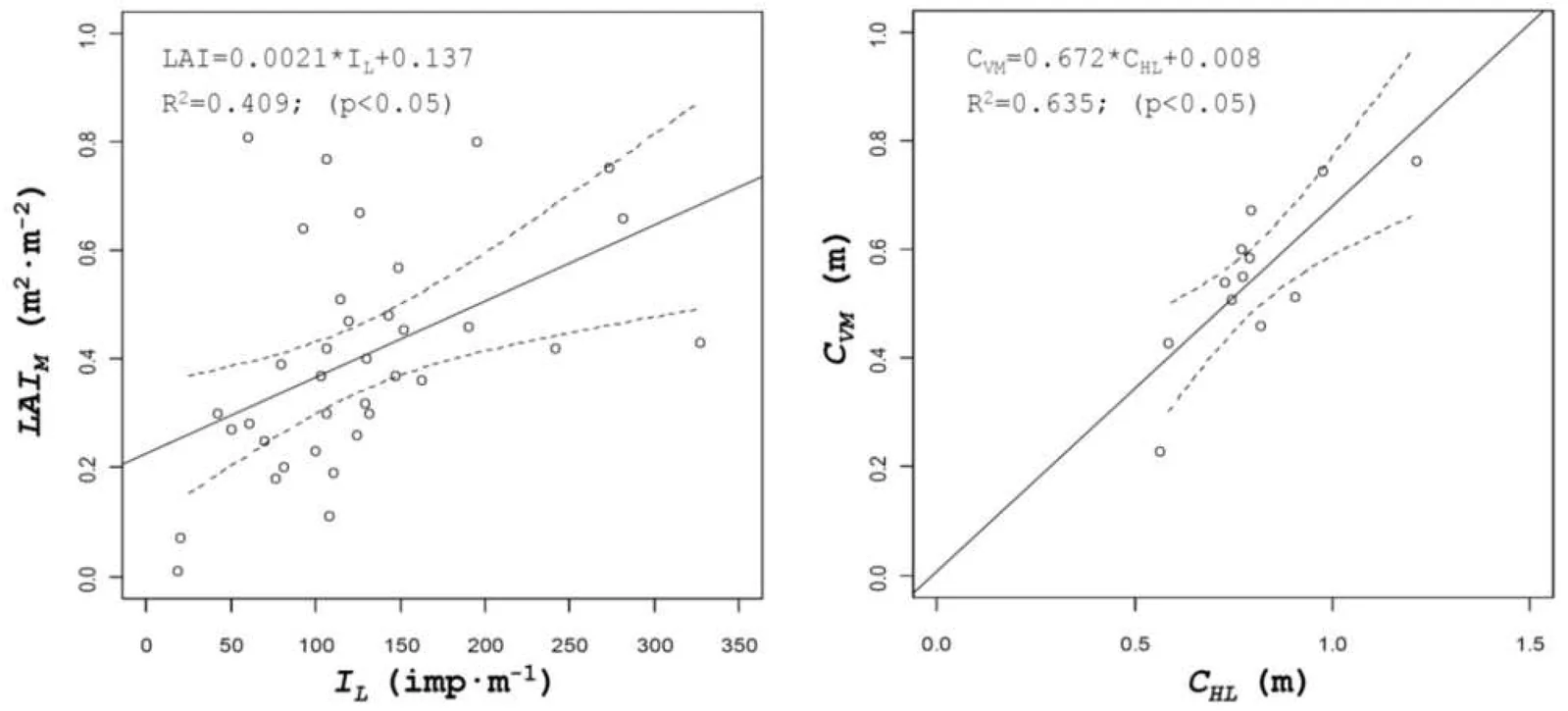
Relationships between LiDAR-derived canopy descriptors and structural parameters in vineyards. **Left**: relationship between LiDAR return impacts and leaf area index (LAI). **Right**: relationship between LiDAR-derived canopy height and manually measured canopy volume. (Reprinted from Ref. [34] under the Creative Commons Attribution 4.0 International license (CC BY 4.0)).

With the development of orchard perception technologies, research attention has gradually shifted from external canopy geometric profiles to the perception of internal structural characteristics. Mahmud et al. constructed a canopy density distribution model using LiDAR point clouds and achieved zonal quantification of canopy density, providing a perceptual basis for sectional nozzle control [14]. Earlier sensor-based work by Llorens et al. showed that LiDAR return impacts were related to leaf area index (LAI), illustrating the potential of LiDAR return statistics as a proxy for foliage abundance [34], as shown in Figure 2. Jiang et al. further proposed a variable-rate spraying method based on foliage area volume density (FAVD) in which internal leaf distribution characteristics were directly used for liquid-flow-rate decision making. By establishing a mapping relationship between FAVD and nozzle PWM control, precise spray control oriented toward canopy structural characteristics was achieved [46]. These studies indicate that internal structural parameters, such as canopy density and foliage area volume density, can more realistically reflect the spatial distribution of spray deposition targets and are gradually becoming important decision-making variables in variable-rate spraying systems. From a regulation perspective, FAVD and leaf area density (LAD) mainly support liquid-flow-rate allocation by characterizing foliage abundance and distribution, whereas porosity and permeability are more closely related to airflow-assisted regulation because they influence airflow propagation and droplet penetration within the canopy.

Porosity- and permeability-related structural information is particularly important for airflow-assisted regulation. Hu et al. proposed a point-cloud-based multidimensional prescription map approach for variable-rate spraying. In their study, long short-term memory (LSTM) was used to separate leaf and woody point clouds, optimize nozzle layout, and divide spraying units. Leaf density, depth information, and an airflow velocity attenuation model were then integrated to calculate spray volume and air velocity, while hardware-in-the-loop tests were conducted to optimize the control strategy. The experimental results showed that uniform deposition could be maintained even in sparse or overlapping canopy regions, while pesticide use was reduced by approximately 42% compared with conventional continuous spraying. These results suggest that internal structural information may provide an important basis for nozzle parameter optimization and local spray-zone division [28].

Nevertheless, the engineering acquisition and stable representation of internal structural information still face considerable challenges. On the one hand, such information often depends on high-quality three-dimensional reconstruction or depth estimation, which is susceptible to occlusion, illumination variation, and insufficient sampling density. On the other hand, there is still a lack of unified, reliable, and broadly applicable mechanistic models for stably mapping structural parameters, such as local density, porosity, and permeability, into specific spray-rate and airflow control variables. Therefore, although internal structural information has higher theoretical value for spray control, its universality and operational feasibility in engineering applications still need to be further improved.

### 2.3. Canopy Status and Prescription-Related Information

As the objective of orchard spraying shifts from simple structural matching toward precision pest and disease management, canopy status and prescription-related information become increasingly important. Such information generally includes the spatial distribution of pests and diseases, leaf health status, fruit location, growth variability, and the derived spraying priority or prescription level. Compared with geometric profile or internal structural information, this level of information is more directly related to decision-making questions such as “where spraying is more needed” and “how much spray is more appropriate”. It therefore represents an important transition of orchard spraying systems from structure-driven regulation to demand-driven regulation.

**Figure 3:**
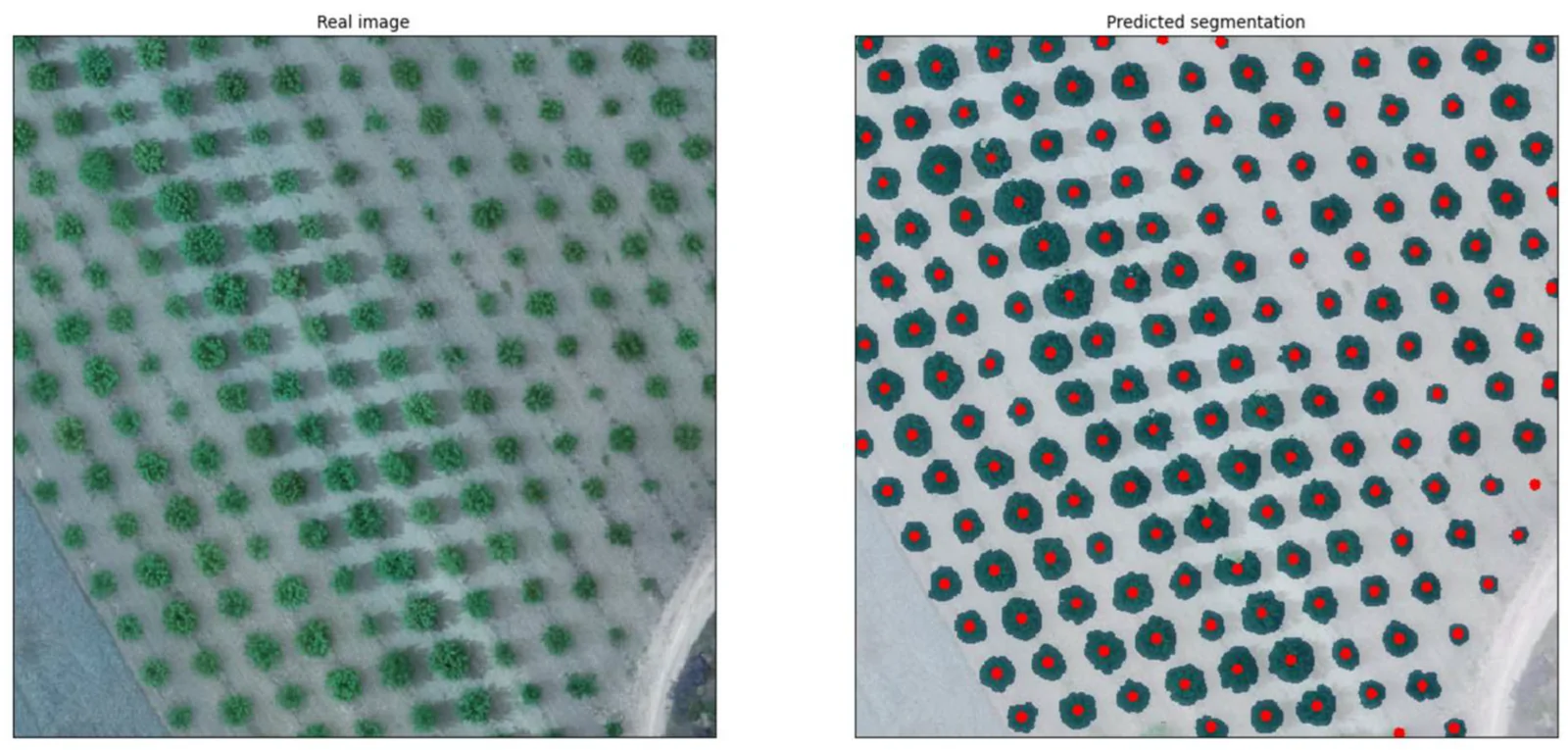
Orchard-scale canopy mapping based on UAV imagery and semantic segmentation. The left panel shows an undersampled orchard orthomosaic with large- to medium-sized canopies, and the right panel shows the segmentation predicted by the model. (Reprinted from Ref. [50] under the Creative Commons Attribution 4.0 International license (CC BY 4.0)).

In existing studies, machine vision and depth sensing have progressively connected canopy status recognition with spray prescription generation. Tree-level systems can integrate target detection, canopy-density classification, fruit detection, and spatial summarization to support localized management [47]. Disease-aware spraying provides a more direct perception-to-actuation link: Zhang et al. classified pear lesion severity and adjusted nozzle PWM duty cycle accordingly, reducing spray volume by 51.9% while maintaining spray quality [22]. Targeted vision systems have also localized diseased leaves for site-specific treatment [48], while attention-enhanced flower-density detection illustrates how biologically meaningful target density can be converted into a spatial treatment-demand variable [49]. Orchard-scale UAV studies further support vigor, flower/fruit, and health mapping [50,51]. Figure 3 illustrates a representative example of UAV-based orchard canopy mapping through semantic segmentation. These examples show that status perception is most valuable when the output is spatialized and explicitly tied to treatment priority or dosage rather than reported only as a recognition accuracy.

At the spray execution level, status-related information is commonly transformed into prescription layers that map disease severity, vigor, fruit or flower distribution, and treatment priority to spatially distributed nozzle or dosage commands. However, recognition confidence alone is insufficient: a usable prescription also requires georeferencing, a defined treatment threshold, spatial resolution compatible with the actuator footprint, and timing consistent with platform motion. This distinction is important because the scientific question is not simply whether disease or vigor can be detected, but whether the detected variation changes a spray decision in a reproducible and agronomically defensible way.

### 2.4. Spatial-Geographic and Operational-Context Perception

Coordinated spraying requires not only knowledge of what the canopy is but also of where the target is relative to the sprayer and when an actuator command must be executed. Orchard mapping, tree row and individual tree localization, platform–tree registration, and motion-state estimation therefore form a fourth perception-information class. INS/GNSS-corrected LiDAR has been used to compensate canopy point clouds on uneven terrain [15], while LiDAR-SLAM orchard reconstruction provides a route to tree-level mapping in environments where repeated geometric structures complicate localization [52]. At the prescription-map scale, RTK positioning can register precomputed spatial dosage commands to the moving platform [53]. These outputs directly determine spray-zone entry and exit, delay compensation, trajectory adjustment, and whether the correct tree or canopy sector receives the intended dose.

Operational context also includes platform attitude, obstacle/terrain constraints, and disturbances that can shift the spray footprint. Millimeter-wave radar or related range sensing can provide robust target/obstacle cues under adverse visual conditions [54]. For aerial and boom-based spraying platforms, obstacle-avoidance requirements, liquid-tank-induced attitude changes, and roll disturbances are not canopy descriptors, but they are relevant because they propagate into spatial registration and spray-placement errors [55,56]. In this review, these studies are retained only to clarify the operational-context layer of the perception–actuation chain; they are not treated as evidence for canopy-parameter perception itself. A complete coordinated-control architecture should, therefore, maintain a common spatial reference connecting target geometry/status, platform pose, actuator location, forward speed, and total sensing–decision–actuation delay.

## 3. Perception Technologies as Inputs to Coordinated Regulation

Building on the information categories defined in Section 2, this section compares the sensing principles, information-acquisition capabilities, and operating suitability of representative perception technologies. Application examples are retained only to clarify the control relevance of the acquired information.

### 3.1. LiDAR Sensing for Structural and Spatial-Registration Inputs

LiDAR is one of the most representative sensing technologies currently used for orchard canopy structural modeling. Its major advantage lies in its ability to actively acquire three-dimensional point-cloud information without relying on natural illumination. Therefore, it can maintain relatively stable geometric measurement performance under shaded, backlit, and low-light conditions. This makes LiDAR particularly advantageous for canopy volume estimation, profile reconstruction, hierarchical structure analysis, and spatial position measurement, especially in orchard canopy perception tasks involving complex structures, variable illumination conditions, and medium- to long-range operating scenarios. Laser scanning has also been evaluated for detecting complexly shaped plant targets specifically for variable-rate sprayer development [57]. Reviews and sensor-development studies consistently identify ultrasonic, LiDAR, and other electronic canopy sensors as foundational technologies for site-specific tree-crop application, with canopy-width-proportional control representing an early implementation pathway [58,59,60,61].

**Figure 4:**
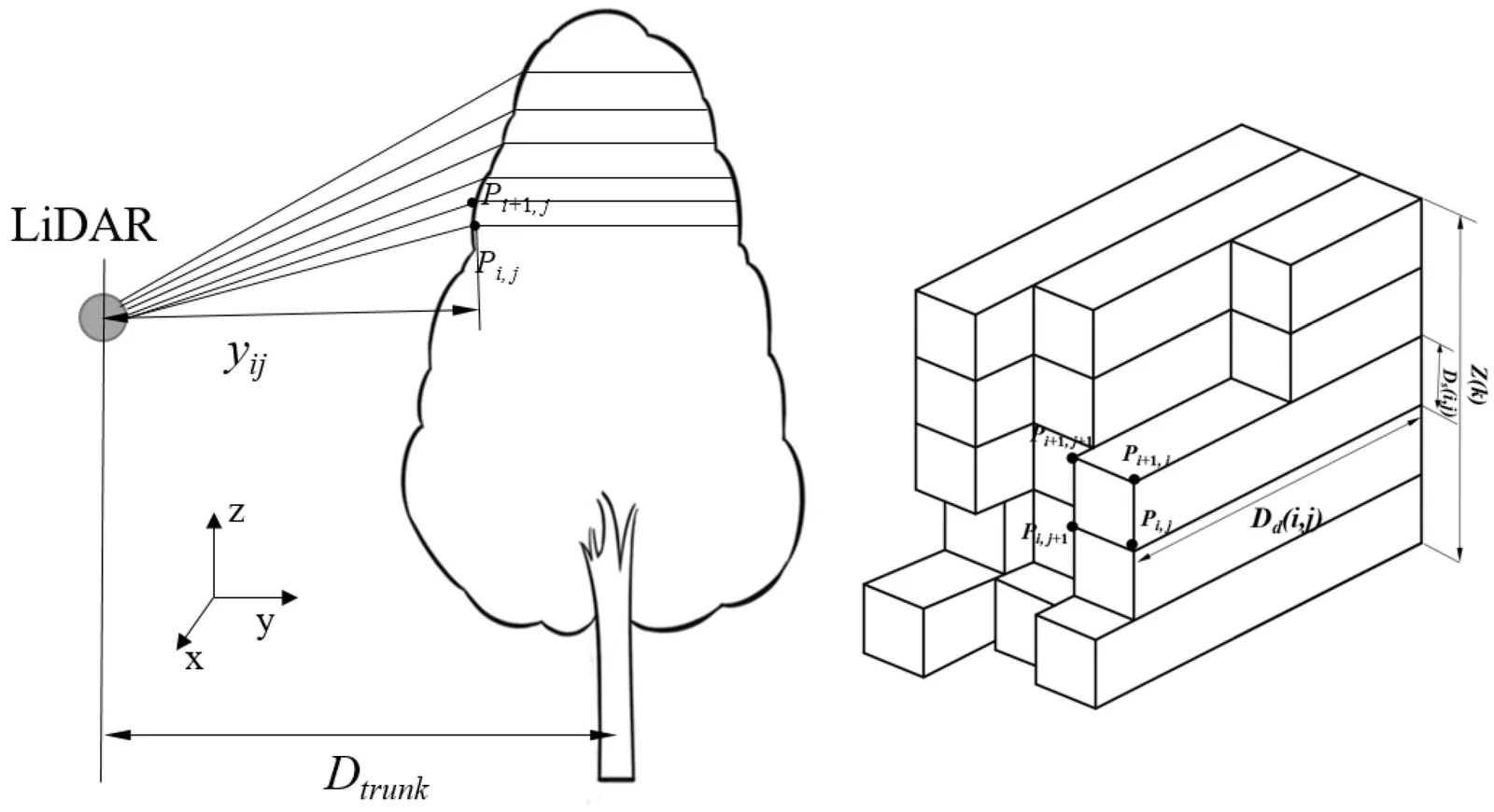
Schematic of the LiDAR-based canopy volume detection algorithm used for variable-rate orchard spraying. (Reprinted from Ref. [62] under the Creative Commons Attribution 4.0 International license (CC BY 4.0)).

From the perspective of coordinated spray regulation, LiDAR is particularly suitable for serving as a primary structural sensor. Zhang et al. developed an orchard variable-rate spraying system based on LiDAR-derived canopy volume measurement. In this system, real-time canopy volume information was converted into spray demand and a solenoid-valve PWM duty cycle, and a speed-adaptive delayed spraying mechanism was integrated to achieve coordinated matching between canopy structural perception and nozzle actuation control. The canopy volume estimation error, average liquid-flow-rate control error, and system response time were 2.84%, 8.78%, and 160 ms, respectively, indicating that LiDAR can be directly used as an input source for variable-rate spray control [62]. A representative LiDAR-based canopy-volume detection algorithm is shown in Figure 4. Mahmud et al. addressed the problem that LiDAR point clouds are susceptible to vehicle attitude variations on uneven orchard terrain. By integrating 3D LiDAR with an inertial navigation system/global navigation satellite system (INS/GNSS), they corrected the spatial positions of canopy point clouds under sloped terrain conditions, thereby improving the accuracy of canopy density measurements and reducing the potential risk of off-target spraying caused by perception–position offsets [15]. These studies indicate that LiDAR can not only extract structural parameters such as canopy volume, thickness, and density, but also be integrated with speed sensing, attitude perception, and nozzle actuation control. Therefore, LiDAR provides reliable support for sectional nozzle switching, variable liquid-flow-rate regulation, and target correction under complex terrain conditions, and it represents an important structural perception basis for current coordinated orchard spraying systems. Representative variable-rate spraying technologies based on LiDAR sensing are summarized in Table 2.

However, LiDAR still has several limitations in engineering applications. First, the relatively high equipment cost restricts its widespread deployment in small- and medium-sized orchards and on low-cost operating platforms. Second, under wind-induced leaf motion, local occlusion, and dynamic field operation conditions, point-cloud data may suffer from motion artifacts, sparsity, holes, and local missing regions, thereby affecting the stability of structural feature extraction. Third, LiDAR has limited capability in semantic recognition, and the identification of fruits, lesions, and fine target regions still requires the support of other sensing modalities. Therefore, although LiDAR is well suited to play a dominant role in orchard canopy structural perception, it is difficult for LiDAR alone to complete all perception tasks required for complex spray control.

### 3.2. Vision Sensing for Semantic and Near-Field Geometric Inputs

Compared with LiDAR, the greatest advantage of vision sensing lies in its ability to acquire rich color, texture, and semantic information at relatively low cost. RGB imaging is well suited to canopy segmentation, disease-region recognition, and other semantic tasks; RGB-D and binocular systems additionally provide depth for near-field localization, profile reconstruction, and canopy-volume estimation. To keep the scope focused, the revised review removes harvesting-, grading-, and cultivar-identification studies that do not establish a direct spray-control or spatial-registration linkage. A trunk-target detection study is retained as a transferable example only where tree localization is explicitly relevant to platform–tree registration [67].

Existing studies have demonstrated the applicability of vision-based methods in supporting spray control. Cong et al. achieved citrus canopy instance segmentation using RGB-D images and an improved Mask R-CNN model, in which depth information was used to suppress interference from complex backgrounds, thereby providing a basis for target canopy recognition in variable-rate spraying [17]. Mahmud et al. combined UAV multispectral imagery with ground RGB images and used vegetation indices and Mask R-CNN to detect and segment apple fire blight-infected regions, supporting disease target localization and precision management [18]. These studies illustrate the complementary roles of different visual modalities: RGB imaging primarily supports semantic target recognition and disease-region segmentation, whereas RGB-D sensing can use depth information to improve near-field localization and geometric characterization. Representative vision-based orchard target perception methods supporting precision spraying are summarized in Table 3. Depth-assisted localization has likewise been demonstrated for apple targets using RGB-D imaging and for potted flowers using a ZED stereo camera, reinforcing the value of depth information for near-field target localization [68,69].

**Figure 5:**
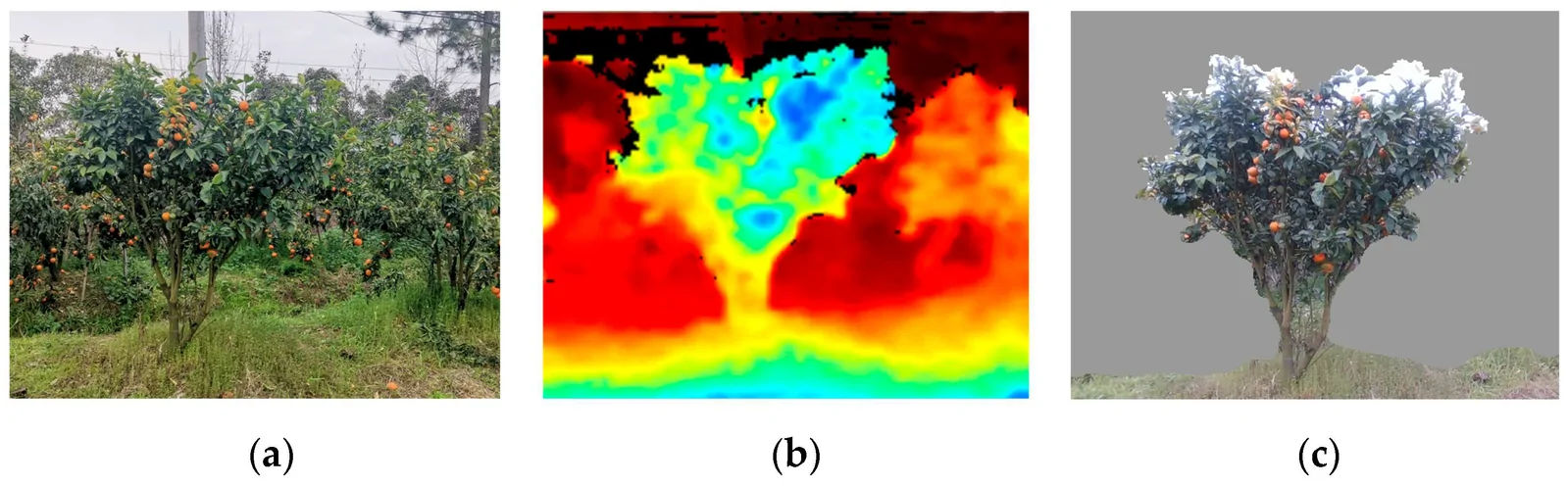
RGB-D image generation for citrus crown perception: (**a**) RGB image; (**b**) depth image; (**c**) aligned RGB-D image. (Reprinted from Ref. [17] under the Creative Commons Attribution 4.0 International license (CC BY 4.0)).

A representative RGB-D crown-image generation process is shown in Figure 5 [17], illustrating how aligned color and depth data can support near-field canopy perception. In coordinated spray regulation, the most direct value of vision perception lies in local target-oriented control. Fruit detection can provide a basis for priority coverage, lesion recognition can support locally intensified pesticide application, and canopy semantic segmentation combined with three-dimensional volume perception can further generate spray-control parameters. Zhang et al. developed a canopy-volume extraction system for plant-protection UAVs based on binocular vision, enabling real-time estimation of fruit tree canopy profiles and volume and providing a dosage decision basis for precision UAV spraying [16]. Gao et al. integrated RGB-D imaging, YOLO-based instance segmentation, GPU acceleration, and PWM control to develop a variable-rate spraying system for vineyards. This system achieved dynamic spray-flow regulation based on canopy volume and reduced plant-protection chemical consumption by 57.4% [21]. In these applications, binocular vision derives canopy depth from stereo geometry, whereas RGB-D sensing provides synchronized depth data through an integrated depth-sensing unit; both can transform segmented canopy regions into three-dimensional profiles and volume estimates for liquid-flow-rate control. Accordingly, depth-enabled vision extends conventional RGB-based recognition toward three-dimensional canopy quantification and closed-loop spray actuation.

RGB-D sensing and binocular vision are both suitable for near-field geometric perception, although their depth-acquisition mechanisms and operating characteristics differ. RGB-D sensing is advantageous for close-range platforms, low-canopy orchards, and trellised systems because it provides aligned texture and depth information for local thickness estimation, boundary recognition, and layer-specific regulation. However, its stability may decrease under strong illumination and at longer working distances. Binocular vision reconstructs depth from paired images and is suitable for canopy-profile reconstruction and volume calculation when sufficient image texture and reliable stereo calibration are available. Therefore, RGB imaging is more appropriate as a semantic primary sensor, whereas RGB-D and binocular vision can serve as near-field geometric sensors supporting canopy-volume estimation and spray regulation.

### 3.3. Multispectral Sensing for Treatment-Demand Inputs

Different from LiDAR and machine vision, which mainly focus on canopy geometric structure and apparent features, multispectral sensing can reveal leaf health status, water conditions, nutritional differences, and pest and disease distributions from the perspective of spectral responses. Therefore, it has unique value in precision plant protection. For orchard spraying systems, the significance of this technology does not lie mainly in canopy shape reconstruction, but in its ability to supplement physiological and pathological information that is difficult to obtain using conventional structural sensing. This provides support for the transition of spray decision making from “structure matching” to “demand matching”. If LiDAR and vision sensing are more capable of answering questions such as “where trees are located”, “where leaves are distributed”, and “how large the canopy is”, multispectral and hyperspectral sensing have greater potential to answer more management-oriented questions, such as “where spraying is more needed”, “which areas have higher risk”, and “which parts should be prioritized for treatment”.

**Figure 6:**
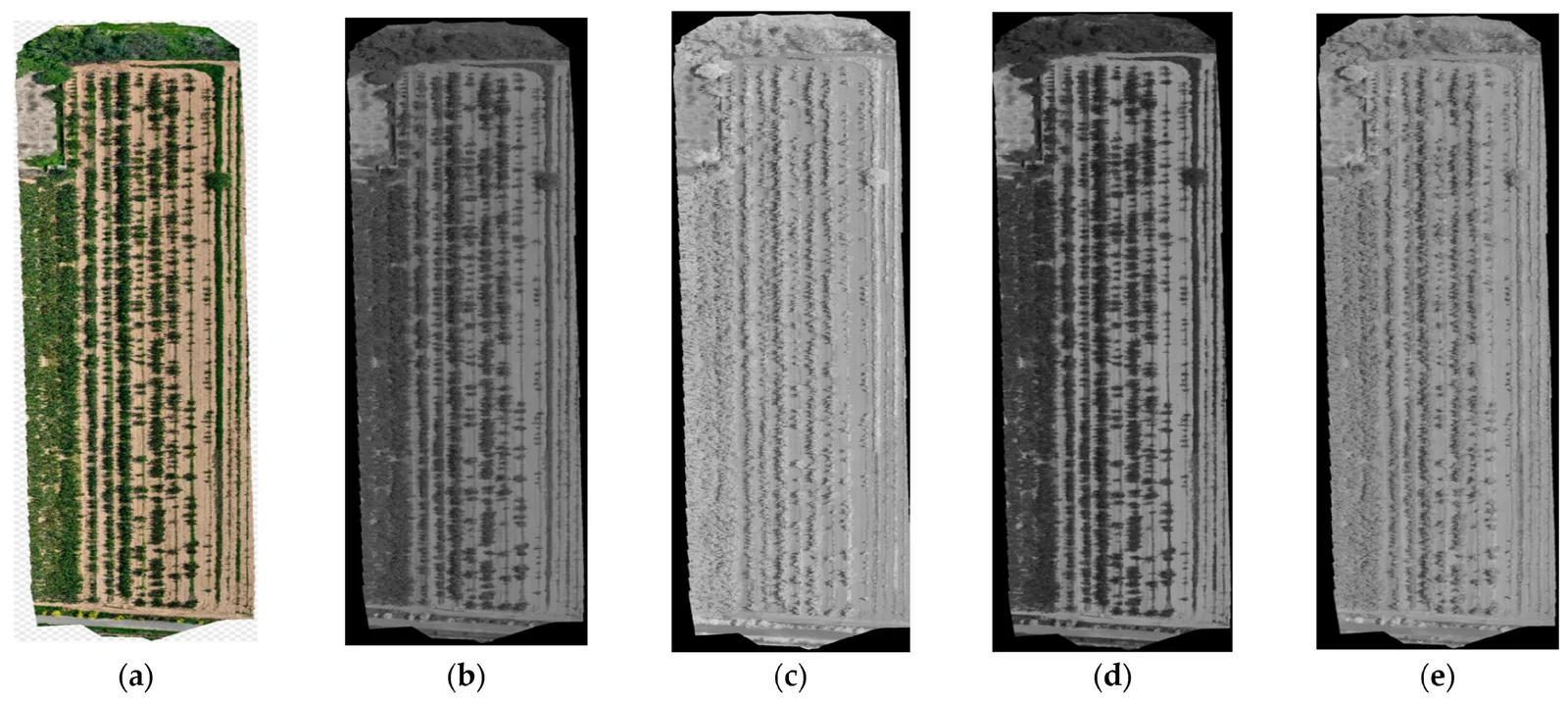
Reconstructed multispectral images of apple trees at the fruit-coloring stage: (**a**) RGB; (**b**) green; (**c**) NIR; (**d**) red; and (**e**) red edge. (Reprinted from Ref. [78] under the Creative Commons Attribution 4.0 International license (CC BY 4.0)).

From the perspective of coordinated regulation of sprayers, spectral information is most valuable when it resolves spatial differences in treatment demand. Orchard and perennial-crop spectral studies demonstrate how multispectral or hyperspectral features can quantify canopy physiological status and disease-related variation [73,74]. A pesticide-residue-sensing study is retained only as a transferable example of how spectral signatures can support treatment-risk assessment rather than as direct orchard spraying evidence [75]. Representative orchard-scale multispectral status-mapping studies are summarized in Table 4. This distinction prevents generic spectral phenotyping from being interpreted as direct validation of orchard spraying decisions. Vegetation-selective spectral sensing and thermal disease imaging are retained as transferable examples showing how spectral or thermal contrast can be converted into treatment-relevant target information [76,77]. Figure 6 presents representative reconstructed RGB and multispectral images of apple trees [78].

However, multispectral and hyperspectral sensing technologies still face constraints in real-time engineering applications. High-dimensional spectral data require complex processing, while equipment cost and dynamic field conditions limit stable online deployment. Moreover, quantitative mappings from spectral features and prescription levels to actuator commands remain insufficiently developed. At the current stage, offline prescription-map generation is more mature; multispectral/hyperspectral sensing is, therefore, better suited to offline prescription-map generation, priority-region identification, and demand-level classification than to direct real-time actuator control. With advances in lightweight sensors, rapid feature extraction, and online decision models, these technologies may play a more direct role in real-time demand-matched spraying.

## 4. Coordinated Regulation Mechanisms and Applications in Orchard Variable-Rate Spraying

Section 2 and Section 3 provide the control-relevant perception inputs. This section is the analytical core of the review and focuses on how those inputs are converted into executable nozzle, liquid-flow, airflow, spatial, and timing commands and on how multiple actuator variables are coordinated during orchard variable-rate spraying.

### 4.1. Canopy-Perception-Based Liquid-Flow Regulation for Variable-Rate Spraying

Compared with nozzle on–off control based on the simple judgment of canopy presence or absence, variable liquid-flow-rate regulation better reflects the core objective of precision pesticide application. Specifically, under the premise of ensuring effective deposition in target regions, the liquid flow rate per unit canopy space should be dynamically matched according to canopy structure, spatial distribution, and operational status. Existing studies have shown that canopy volume, vertical layer distribution, branch and leaf density, and platform forward speed are important variables affecting liquid-flow-rate allocation. Fundamentally, this is because the leaf-area carrying capacity, droplet penetration resistance, and pesticide demand intensity vary among different canopy spatial units. Orchard dose-model comparisons confirm that canopy-dependent dose selection affects deposition, while transferable variable-rate systems in other crops show the feasibility of target-dependent application and closed-loop spray-quantity regulation [83,84,85]. European orchard and vineyard prototypes demonstrated continuous canopy-adaptive flow control, while laser-scanning systems further synchronized nozzle output with measured tree structure [86,87,88,89].

In this direction, a technical pathway has gradually emerged from binary target detection toward progressively richer perception-to-actuation mappings. LiDAR-based systems first linked canopy profile or volume to PWM dosage and spray-zone control [29]. In the original study by Liu et al., this configuration was described as a LiDAR-based pitch variable-rate sprayer using a pitch-spray mechanism control model: an improved α-shape algorithm estimated canopy volume, a PWM dosage model determined liquid delivery, and the orientable spray mechanism adjusted the spray trajectory to fit the canopy contour [29]. Layer-specific methods then allocated different liquid flow rates to vertically resolved canopy units according to local volume or density [90]. Vision-based prototypes additionally demonstrated real-time target-zone control using depth or stereo information [91].

**Figure 7:**
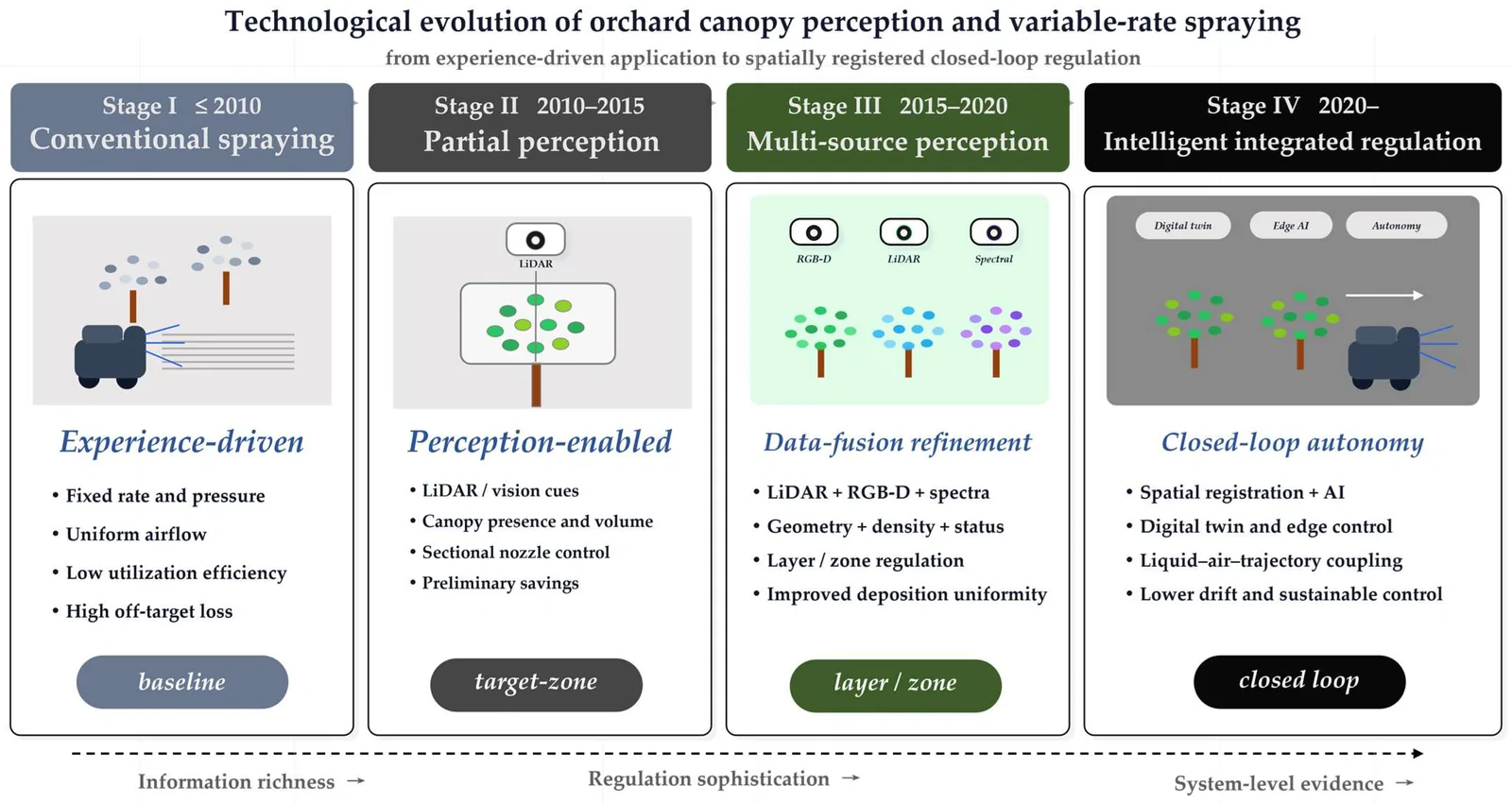
Technological evolution of orchard variable-rate spraying from target-presence detection to geometry-based dose matching, layer- and zone-specific regulation, and spatially registered prescription-driven coordinated control. The corresponding perception-to-actuation mapping progressively evolves from binary switching toward coupled liquid–air–trajectory regulation.

Figure 7 synthesizes this technological progression from target-presence detection to increasingly integrated perception-to-actuation regulation. Table 5 summarizes representative orchard systems that establish a direct link between perception and variable-rate actuation. Transferable control architectures from laser-guided greenhouse spraying and vision-based row-oriented/dual-face target spraying are retained only to illustrate actuator-timing and target-zone control strategies that can be adapted to orchard platforms [92,93,94].

Overall, liquid-flow regulation in variable-rate spraying has gradually evolved from early “adjustment according to overall tree size” toward “regulation according to local canopy structure, layer-specific differences, and target distribution”. This evolution improves the matching degree between liquid delivery and actual canopy demand, helping to reduce pesticide application in non-target regions, decrease pesticide waste, and improve deposition performance in complex canopies. However, these methods place higher demands on perception accuracy, real-time processing, actuator response, airflow organization, and motion-delay compensation. In mobile spraying, actuation timing should be corrected according to the sensor–nozzle distance, forward speed, valve response time, and computation delay; otherwise, spatial misalignment may cause local over- or under-application. Therefore, highly robust, multi-layer, and adaptive variable-rate spraying control for complex orchard scenarios remains an important development direction for intelligent plant-protection equipment. At the actuator and timing-control level, breakpoint-continuous spraying, single-drive variable-angle spray-mechanism adjustment, staggered-phase PWM, and angle correction based on the effects of spray angle and forward speed on deposition provide practical strategies for improving spatial and temporal spray uniformity [96,97,98,99].

### 4.2. Canopy-Perception-Based Airflow-Assisted Regulation

During orchard spraying, airflow-assisted devices are essential for droplet transport, canopy penetration, and internal deposition, especially in dense or occluded canopies. Liquid atomization alone is often insufficient to deliver droplets effectively into the canopy interior. Proper regulation of air velocity, air volume flow rate, and airflow direction can improve penetration and deposition uniformity, whereas excessive airflow may increase drift and leaf disturbance. In this review, fan speed and outlet area are treated as actuator variables, while air velocity, air volume flow rate, and direction are airflow-control outputs. These quantities should be linked to canopy thickness, density, porosity, and operating conditions rather than inferred from canopy-resistance models developed for evapotranspiration; the latter references have, therefore, been removed. Leaf-motion studies under spray airflow are retained because they directly demonstrate how aerodynamic forcing changes droplet interception and deposition [100,101].

**Figure 8:**
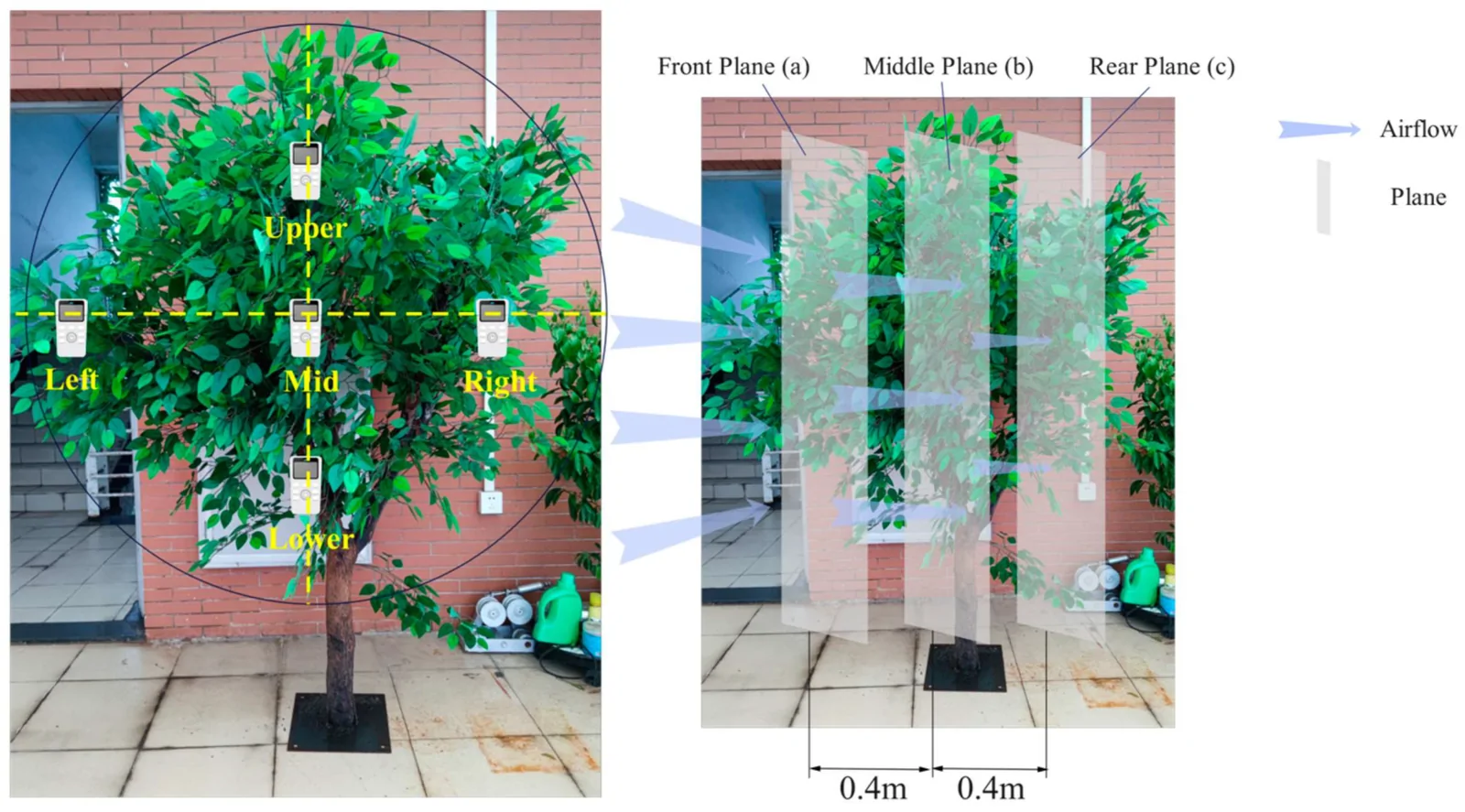
Anemometer layout across three vertical canopy planes (front, middle, and rear) for airflow-field validation of rail-mounted orchard plant-protection equipment. (Reprinted from Ref. [103] under the Creative Commons Attribution 4.0 International license (CC BY 4.0)).

Existing studies indicate that airflow-assisted regulation is shifting from empirical control toward canopy-structure-driven quantitative regulation. Dou et al. designed an air-assisted sprayer that independently adjusted fan speed and outlet area to decouple air velocity and air volume flow rate. They further established regulation models for constant-air-velocity/variable-air-volume flow-rate and constant-air-volume flow-rate/variable-air-velocity conditions [26]. Vigo-Morancho et al. integrated LiDAR-based canopy characterization, multi-point air-velocity measurements, and deposition tests to analyze the effects of fan speed, canopy depth, height, and vegetation density on airflow attenuation and deposition [102]. Xue et al. optimized outlet air velocity, airflow direction adjusted through fan pitch angle, and rail-vehicle forward speed by combining computational fluid dynamics simulations, airflow-field validation, and field experiments [103]. The corresponding anemometer layout across the front, middle, and rear canopy planes is shown in Figure 8. Representative airflow-assisted spraying systems and regulation methods are summarized in Table 6. An autonomous single-track air-assisted sprayer developed for greenhouse production further illustrates engineering integration of airflow assistance, platform motion, and spraying execution [104]. Sprayer calibration and droplet-scale studies further indicate that output air-assist patterns, air velocity, liquid flow rate, and equipment settings should be characterized quantitatively rather than adjusted solely from operator experience [105,106,107,108]. Earlier orchard studies on variable air assistance, precision horticultural air-assist patterns, vineyard airflow, and controllable fan outlets provide complementary evidence that airflow magnitude and distribution must be matched to canopy architecture rather than treated as fixed machine settings [109,110,111,112].

From a mechanistic perspective, airflow regulation and liquid-flow-rate regulation are interdependent. Liquid flow rate determines the supplied liquid mass, whereas air velocity, air volume flow rate, and direction influence whether droplets reach target layers, penetrate canopy gaps, and remain on foliage. Canopy descriptors should, therefore, be converted into coupled liquid–air commands rather than optimized independently. Selected nozzle-scale studies are retained only for specific mechanistic questions: low-pressure jet breakup provides evidence on droplet formation [121], while ultrasonic air-assistance and nozzle-geometry studies illustrate how atomization and trajectory properties can alter spatial deposition [122,123]. These studies are not treated as orchard-system validation. The remaining evidence is strongest for prototype- or canopy-scale demonstrations, while a general model that maps porosity or permeability to a transferable airflow setpoint is still lacking. Additional transferable mechanistic evidence includes numerical validation of droplet deposition on leaf surfaces and electrostatic air-assisted spray distribution [124,125]; these studies are used to interpret deposition mechanisms rather than as orchard-system performance validation.

Overall, canopy-perception-based airflow-assisted regulation has evolved from simple fan-speed gear settings toward coordinated regulation of fan speed and outlet area and quantitative control of air velocity, air volume flow rate, and airflow direction. However, the mapping from canopy structure to these actuator and airflow parameters remains insufficiently mature, and the coupled mechanisms among airflow attenuation, droplet movement, leaf disturbance, and deposition require further investigation. Future studies should integrate LiDAR- or vision-based canopy perception, CFD-based airflow field modeling, online air-velocity feedback, and rapid actuator control to develop adaptive regulation models. This will promote the transition from fixed-air-volume flow-rate assistance toward structure-adaptive and deposition-oriented airflow control. Orchard-specific CFD studies provide a complementary mechanistic basis for this task by resolving sprayer airflow, wind interaction, and deposition across different sprayer configurations [117,118], with subsequent assessment frameworks linking simulated airflow and deposition to experimental validation [119,120].

### 4.3. Prescription-Map-Based Coordinated Control Applications

With the continuous development of orchard perception and control technologies, variable-rate spraying systems are gradually evolving from single-parameter regulation toward prescription-map-driven coordinated system control. A prescription map is not simply the result of target recognition; rather, it transforms canopy spatial structure, growth status, leaf area density, disease risk, spraying units, and actuator parameters into executable spray decision schemes. In this way, spatially coordinated regulation of nozzle switching, liquid-flow-rate allocation, and airflow-assisted parameters can be achieved. Compared with real-time control based on a single sensor, prescription-map-based methods can complete multi-source information fusion, spatial zoning, and parameter optimization before field operation, making them more suitable for planned variable-rate operations in large-scale orchards. At broader operational scales, orchard UAV trials in mango and mountainous pear orchards together with integrated UAV detection, pesticide mixing, and adaptive spraying, highlight the importance of linking remote perception with platform-aware application planning [126,127,128]. Prescription-map implementations in vineyards have further shown how canopy variability can be converted into geostatistical or on-farm spatial application strategies, complementing real-time sensor-driven regulation [129,130,131].

**Figure 9:**
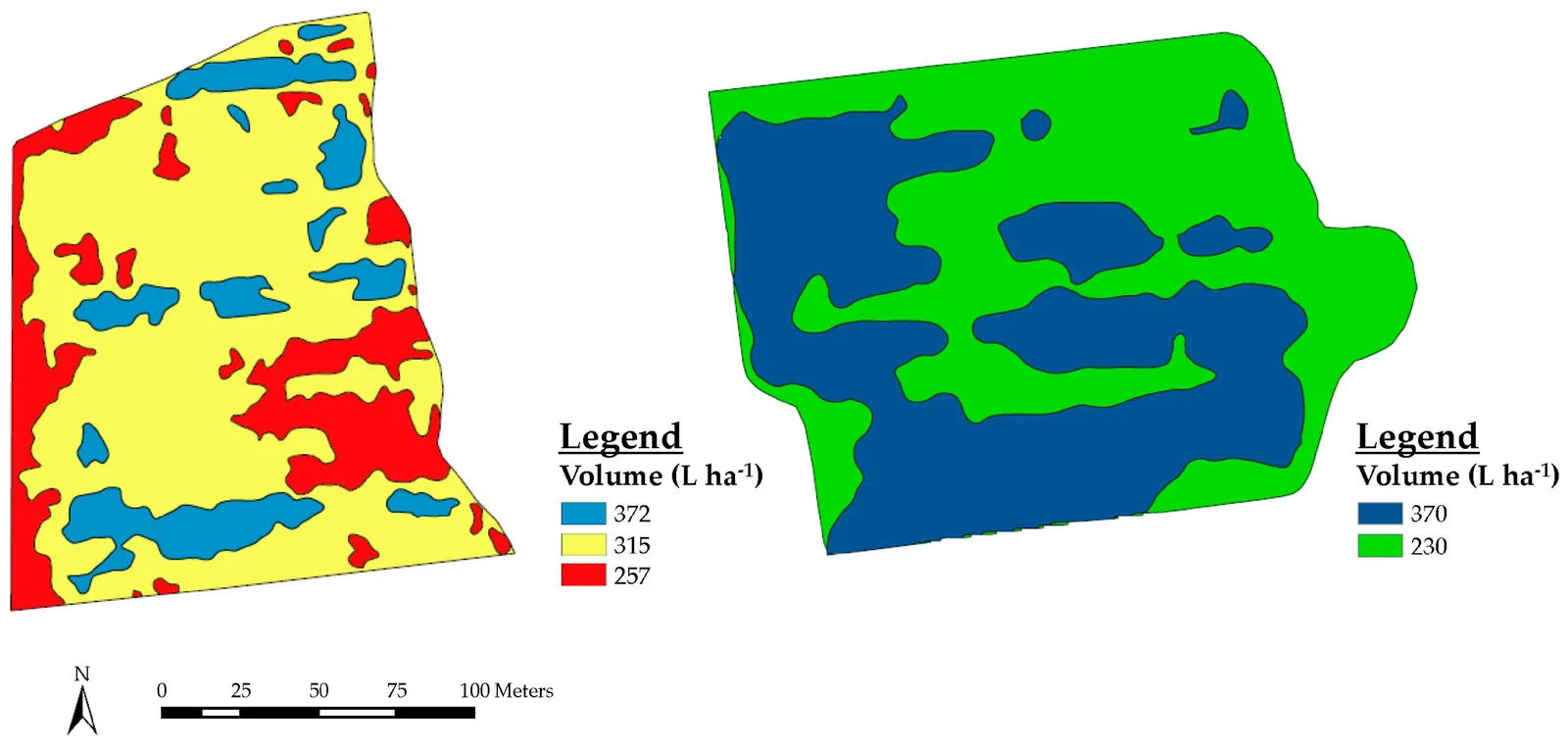
Prescription maps used for field variable-rate pesticide application in vineyards. The maps represent vigor-based spatial zones and their corresponding target application volumes for two field cases. (Reprinted from Ref. [129] under the Creative Commons Attribution 4.0 International license (CC BY 4.0)).

In prescription-map-driven variable-rate spraying, existing studies have promoted the transformation of perception information into actuation control parameters at different spatial scales. Hu et al. developed a multidimensional spraying prescription map based on fruit tree point clouds, integrating leaf–wood separation, nozzle topology optimization, spraying unit division, airflow demand estimation, and dosage control into a unified framework. This enabled coordinated regulation of nozzles, liquid flow rate, and airflow according to spatial differences in canopy structure [28]. Han et al. generated orchard voxel prescription maps using UAV multispectral imagery and three-dimensional reconstruction and combined real-time kinematic (RTK) positioning with PWM nozzle control to achieve offline variable-rate pesticide application. This provided a feasible pathway for low-cost and large-scale variable-rate spraying in orchards [53]. Roman et al. constructed vineyard vigor zoning maps based on multispectral imagery and combined leaf area index (LAI) estimation with the DOSA3D decision system to generate pesticide application prescription maps, thereby achieving spatial-vigor-based zonal dosage management [132]. A representative set of vigor-based prescription maps used for field variable-rate application is shown in Figure 9 [129]. Overall, prescription-map-based methods are promoting the development of orchard spraying from single structural perception control toward multi-source information fusion, spatial zoning decision making, and coordinated actuator regulation. Representative prescription-map-based coordinated control technologies are summarized in Table 7.

Overall, prescription-map-driven coordinated control is promoting the transformation of orchard spraying from “local perception–instant response” toward “multi-source perception–spatial modeling–parameter pre-allocation–actuation control”. Its core advantage lies in its ability to integrate canopy structure, crop status, and spraying actuators into a unified spatial decision-making framework, thereby extending spray control from single-variable regulation to systematic coordinated optimization of nozzles, liquid flow rate, and airflow assistance. However, the engineering application of prescription-map-based methods still faces several challenges, including the cost of high-precision three-dimensional reconstruction, prescription map timeliness, spatial registration errors, actuator response delay, sprayer attitude fluctuation, and model transferability across different crops and tree architectures. Future research should further strengthen multi-source perception data fusion involving UAVs, LiDAR, and machine vision; establish prescription map representation standards oriented toward spraying actuators; and incorporate online feedback correction mechanisms. These efforts will facilitate the transition from offline prescription maps to real-time adaptive coordinated control.

### 4.4. Classification of Perception-to-Actuation Pathways for Coordinated Regulation

**Figure 10:**
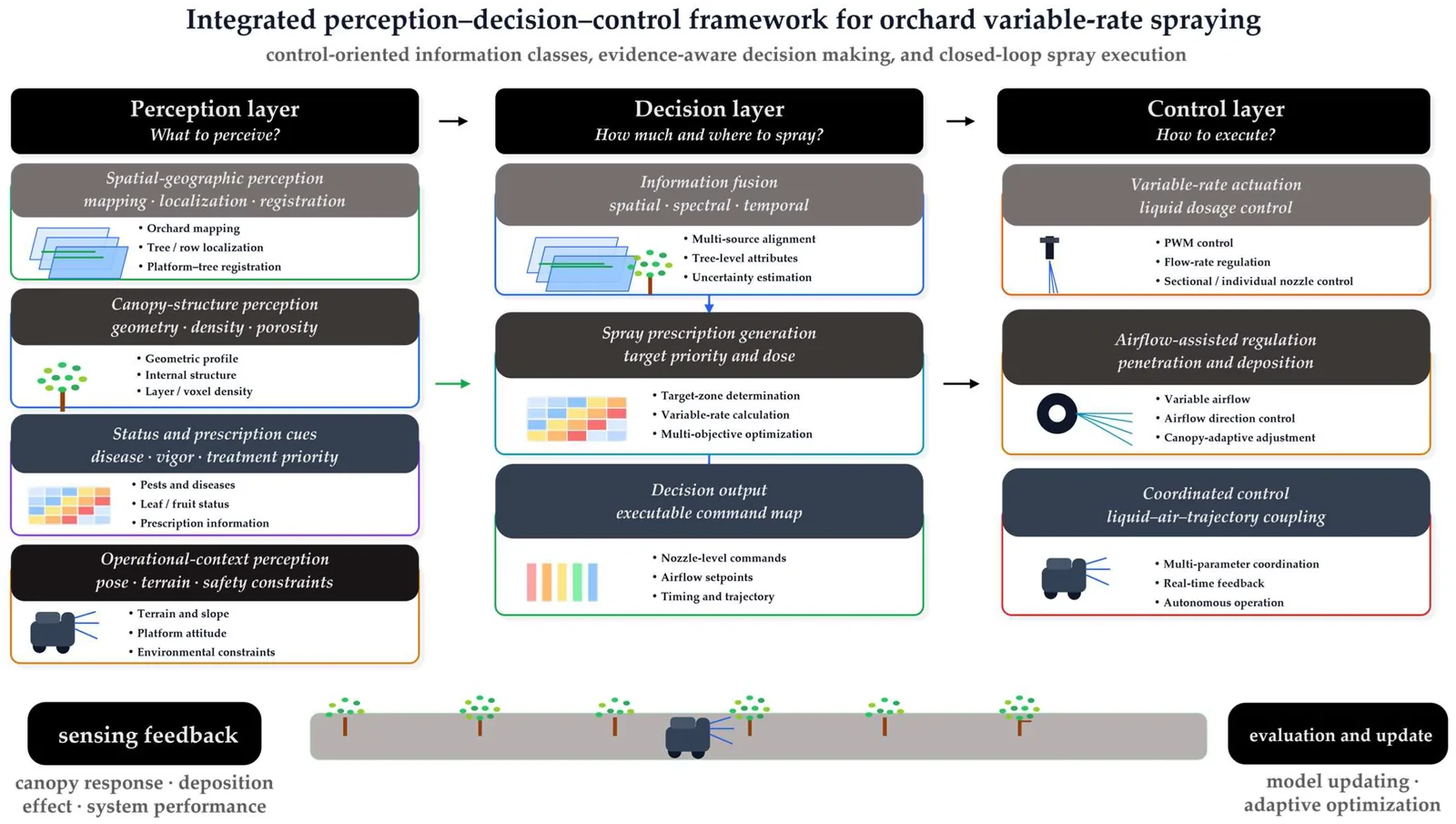
Integrated perception–decision–control framework for coordinated orchard variable-rate spraying. The framework links perception objects, control-oriented information, sensing and spatial registration, perception-to-actuation mapping, and coordinated spray execution. Feedback from deposition and platform state closes the control loop, highlighting the roles of spatial registration and latency compensation in coordinated regulation.

Rather than serving as another study inventory, Table 8 classifies the perception-to-actuation pathway by control-oriented information class and associated regulation mechanism. Geometric information primarily supports target localization, spray-zone definition, and dose matching; internal structural information supports layer-specific liquid-flow allocation and adaptive airflow; status information supports treatment-priority decisions; and spatial-geographic/operational context provides the registration and timing needed to place those commands on the correct tree or canopy sector. Figure 10 summarizes these four information classes within a unified closed-loop architecture.

A cross-study comparison further shows why perception accuracy cannot be interpreted independently of control evidence. Table 9 grades representative studies by validation level and reports only outcomes explicitly stated in the reviewed literature. The strongest evidence is obtained when a perception variable is linked to an actuator command and validated under field conditions with deposition, pesticide saving, control error, or end-to-end response metrics. By contrast, perception-only accuracy or hardware-in-the-loop results provide weaker evidence for agronomic benefit and should not be treated as equivalent to field validation.

## 5. Current Challenges and Future Development Directions

### 5.1. Current Challenges

Although research on orchard canopy perception and coordinated regulation of sprayers has progressed rapidly, several key bottlenecks remain before stable, efficient, and widely transferable engineering applications can be achieved. First, the mapping relationship between perception outputs and actuation parameters remains insufficiently developed. Existing studies can already identify canopy boundaries, estimate canopy volume, characterize local density, and recognize diseased or priority regions with relatively high accuracy. However, the subsequent conversion of these perception results into nozzle switching, liquid-flow-rate levels, spray angles, and fan parameters still relies heavily on empirical thresholds, grading rules, or simplified models. This means that although a system may be able to perceive canopy differences, it may not be able to stably and reasonably convert these differences into optimal control commands. In particular, in scenarios where canopy structure, airflow propagation, and droplet deposition are highly coupled, empirical mapping methods are unlikely to fully translate improvements in perception accuracy into real control benefits.

Second, robustness and generalization ability under complex orchard environments remain insufficient. Different fruit trees vary considerably in tree architecture, branch and leaf density, leaf morphology, and fruit distribution. Even for the same fruit species, canopy structure can change substantially across growth stages, pruning practices, and management intensities. As a result, perception models and control strategies that perform well in a single scenario may show significant performance degradation when transferred across tree species, seasons, or orchards. Meanwhile, orchard environments commonly involve non-uniform illumination, shadow occlusion, leaf reflection, cluttered backgrounds, platform vibration, and natural wind disturbance. These factors further amplify perception errors and actuation deviations, thereby weakening system stability under real operating conditions.

Third, system real-time performance and engineering deployability remain under considerable pressure. Point-cloud processing, image segmentation, spectral feature extraction, and prescription map generation all require computational resources and response time. However, orchard spraying platforms usually operate in continuous motion. Once there is an obvious delay among perception, decision making, and actuation, spray control may become spatially misaligned with the target, thereby affecting deposition performance and pesticide utilization efficiency. This issue becomes particularly critical when multi-source information fusion, local priority determination, and online parameter adjustment are performed simultaneously. Therefore, real-time closed-loop capability remains an important constraint on engineering application. Platform robustness is further affected by obstacle avoidance requirements, liquid-tank-induced UAV stability, and spray-boom roll disturbances, which can propagate into spatial spray misalignment [55,56,136].

Finally, system-level coordinated control frameworks remain incomplete. Many existing studies focus more on optimizing individual components, such as improving perception accuracy, variable liquid-flow-rate control, or local prescription generation. However, insufficient attention has been paid to continuous closed-loop coordination among perception, mechanistic modeling, actuation feedback, and control correction. As a result, current systems can often “see” the canopy, but still struggle to truly “understand” the dynamic relationship between canopy structure and spray deposition, making it difficult to form interpretable, transferable, and highly stable coordinated regulation mechanisms. Therefore, moving from isolated technological breakthroughs toward system-level coordinated optimization remains a core issue that must be continuously addressed in this field.

### 5.2. Future Development Directions

Future work should translate each bottleneck in Section 5.1 into a testable scientific question. First, structural descriptors used for spray control require operational definitions. A key question is how canopy porosity, permeability, foliage area volume density, and layer-wise depth should be measured so that values are comparable across sensors and tree architectures. The expected output is a set of sensor-agnostic structural descriptors with uncertainty bounds and a physically interpretable mapping to liquid and airflow demand. Validation should report repeatability across sensors, cultivars, pruning forms, and phenological stages, together with deposition uniformity and penetration error rather than perception accuracy alone.

Second, end-to-end spatial and temporal alignment should be treated as a control problem rather than as an implementation detail. Future systems should jointly model sensor–nozzle geometry, forward speed and platform attitude, computation time, communication delay, valve–fan response, and target motion. A researchable hypothesis is that explicit prediction and delay compensation can reduce the spatial offset between perceived and sprayed canopy sectors under variable-speed operation. The expected output is a latency-aware registration model or predictor evaluated using total sensing-to-actuation delay, longitudinal/lateral spray-placement error, missed-target rate, and closed-loop spray-rate error during continuous field operation.

Third, perception-to-actuation models should become both physically interpretable and transferable. Rather than fitting crop-specific empirical coefficients only, future studies should combine airflow and droplet-transport physics with data-driven residual learning, using canopy geometry, density, porosity, disease severity, meteorological variables, and platform state as explicit inputs. The expected output is a calibrated model that predicts required liquid flow, airflow, or treatment priority with quantified uncertainty. Cross-orchard validation should use leave-one-cultivar-/season-/site-out protocols and report deposition coverage, coefficient of variation, pesticide saving, drift, and calibration error to determine whether improved perception actually yields improved application performance.

Fourth, benchmark datasets and digital-twin-assisted validation should be designed around the complete closed loop. A useful benchmark should synchronize raw sensor data, tree-level geolocation, canopy structure/status labels, platform trajectory, actuator commands, and measured deposition/drift outcomes. Digital twins can then be used for virtual commissioning and hypothesis testing, but they should be accepted only after field calibration against canopy deformation, airflow attenuation, and deposition measurements. The expected output is an open, multi-season benchmark and a reproducible evaluation protocol. Core metrics should include perception uncertainty, spatial registration error, end-to-end latency, actuation tracking error, deposition uniformity, penetration, off-target loss, pesticide saving, energy use, and robustness across tree species and seasons. These concrete outputs would allow future studies to move from isolated module optimization to evidence-based comparison of complete perception–decision–actuation systems.

## 6. Conclusions

This review examined how orchard perception information can be transformed into coordinated regulation commands for variable-rate sprayers. The evidence was organized around four control-oriented information classes—geometric profile, internal canopy structure, crop-/plant-protection status, and spatial-geographic/operational context—and their relationships with LiDAR, RGB/RGB-D and binocular vision, multispectral/hyperspectral sensing, positioning, and multimodal fusion. Geometric and structural information supports target zoning, liquid allocation, and airflow regulation; status information supports demand-oriented treatment; and spatial-geographic/operational perception provides the registration and timing needed to execute these commands on the correct target. No single sensing modality satisfies all control requirements, making multimodal and spatially registered perception increasingly important.

The cross-study synthesis further indicates that the central scientific challenge is not canopy perception accuracy alone, but the reliable, interpretable, and latency-aware conversion of heterogeneous perception outputs into executable liquid-flow, airflow, nozzle, and trajectory parameters. Quantitative evidence is strongest for selected canopy-volume/density and disease-aware spray-rate systems, whereas transferable airflow-demand mappings and cross-season dose models remain comparatively weak. Accordingly, future progress should be judged by end-to-end field outcomes—deposition, pesticide saving, drift, spatial placement, and response latency—rather than by sensing metrics in isolation.

This review is limited by substantial heterogeneity among crop species, canopy architectures, sensing configurations, control variables, platforms, and validation protocols. The available evidence therefore supports a structured narrative synthesis and evidence grading rather than formal meta-analysis. The review explicitly distinguishes orchard-specific evidence from transferable non-orchard methods and retains the latter only when their relevance to sensing, registration, or spray-transport mechanisms is clear. Standardized datasets and evaluation protocols, physically interpretable perception-to-actuation models, and continuous closed-loop field validation are required before results can be generalized confidently across orchards, seasons, and spraying platforms.

## Figures and Tables

**Table 1:** Classification of representative control-oriented canopy perception methods supporting orchard variable-rate spraying.

Method Category	Representative Sensing/Control Strategies	Control-Oriented Outputs/Applications	Refs.
UAV-based canopy mapping and crown extraction	UAV RGB/multispectral imagery, DSM–DTM products, and segmentation/detection models (e.g., SAM and YOLO variants)	Tree geometry, crown localization, prescription zoning, and path planning	[13,35]
Ground-based image segmentation for target-zone recognition	CNN semantic/instance segmentation using FCN, PP-LiteSeg, STDC, and YOLO-based models	Canopy-size classification, target-zone identification, and reduction in non-target spraying	[36,37]
Active-ranging geometry sensing	Ultrasonic, laser, and LiDAR measurements of canopy width, boundary, and volume	Canopy geometry for nozzle switching and electronic variable-rate control	[33,34,38,39,40,41]
Depth-enabled 3D perception linked to dosage control	RGB-D or binocular depth sensing, 3D volume estimation, and PWM actuation	Near-field geometry, canopy-volume estimation, and liquid-flow-rate regulation	[16,21]

**Table 2:** Classification of structural sensing and modeling methods supporting coordinated spray regulation.

Method Category	Representative Sensing/Control Strategies	Control-Oriented Outputs/Applications	Refs.
LiDAR point-cloud structural quantification	Point-cloud density analysis, leaf-area-density estimation, and sensor-placement optimization	Canopy density and foliage descriptors for section-specific regulation	[14,63,64]
Structure-to-airflow/deposition modeling	LiDAR-derived thickness/leaf-area descriptors combined with airflow-loss or deposition models	Airflow-demand estimation and deposition prediction	[25,65]
Ultrasonic canopy-size/density sensing	Ultrasonic sensing combined with tree-row volume and density correction	Online canopy-structure-based liquid-flow-rate regulation	[66]
Pose-compensated structural perception	LiDAR integrated with INS/GNSS, forward-speed sensing, and delayed actuation	Spatially registered canopy information and aligned variable-rate actuation	[15,62]

**Table 3:** Classification of vision-based methods according to their control-oriented outputs for orchard spraying.

Method Category	Representative Sensing/Control Strategies	Control-Oriented Outputs/Applications	Refs.
UAV crown detection and localization	Improved YOLOv4 and lightweight U-Net models applied to aerial imagery	Tree/crown localization for spray-zone definition and orchard-scale targeting	[70,71]
Disease and lesion semantic recognition	Binocular-depth-assisted YOLOv5-LDD and YOLOv7-based classification/detection	Diseased-region identification and targeted treatment decisions	[48,72]
Depth-enabled near-field geometry	RGB-D and binocular vision for aligned depth, profile reconstruction, and volume estimation	Three-dimensional canopy descriptors and dosage inputs for closed-loop spraying	[16,17,21]
Multimodal disease localization	Aerial multispectral and ground RGB imagery with segmentation/deep learning	Spatial disease mapping and treatment-priority definition	[18,22]

**Table 4:** Classification of multispectral and multimodal sensing methods according to treatment-demand information.

Method Category	Representative Sensing/Control Strategies	Control-Oriented Outputs/Applications	Refs.
Spectral physiological-status estimation	UAV multispectral features, vegetation indices, machine learning, and Gaussian-process regression	SPAD/chlorophyll and physiological-status estimation across growth stages/species	[73,74,78,79]
Vigor, health, and disease zoning	Multispectral UAS imagery combined with OBIA, random forest, morphology, or vegetation-index analysis	Vigor classes, diseased-tree identification, and treatment-priority zones	[50,51,80,81]
Multimodal structure–status integration	UAV multispectral imagery combined with point clouds or 3D reconstruction	Integrated canopy-parameter maps and prescription generation	[35,53,82]
Transferable treatment-risk sensing	Spectral or thermal target contrast used to infer risk or treatment demand	Treatment-priority or risk mapping rather than direct actuator validation	[75,76,77]

**Table 5:** Classification of perception-to-actuation methods for target-oriented and variable-rate orchard spraying.

Method Category	Representative Sensing/Control Strategies	Control-Oriented Outputs/Applications	Refs.
Geometry-to-liquid-flow mapping	LiDAR/RGB-D canopy-volume estimation linked to PWM dosage or valve commands	Canopy-matched liquid-flow-rate regulation	[6,21,29,62]
Layer- and zone-specific regulation	Vertical canopy partitioning, local volume/density estimation, and nozzle-specific PWM control	Differentiated liquid delivery among canopy layers or spray zones	[90,91]
Target-zone detection and spatial timing	Radar/vision target detection with zone logic, nozzle timing, and sensor–actuator coordination	Nozzle switching, zone entry/exit control, and timing compensation	[54,91,92,93,94]
Coupled air–liquid regulation	Laser sensing with dual-variable airflow and liquid-delivery control	Coordinated adjustment of liquid flow rate and airflow	[95]

**Table 6:** Classification of airflow-assisted regulation methods for orchard spraying.

Method Category	Representative Sensing/Control Strategies	Control-Oriented Outputs/Applications	Refs.
Fan-speed and outlet-area regulation	Independent fan-speed/outlet-area adjustment, variable air assistance, and controllable fan geometry	Air-velocity and air-volume flow-rate regulation	[26,109,112,113]
Canopy-adaptive airflow regulation	LiDAR/canopy-structure inputs combined with dual-fan, multi-duct, or multi-fan sprayers	Canopy-matched airflow, improved penetration, and reduced off-target drift	[24,102,114,115,116]
CFD- and field-based airflow optimization	CFD modeling combined with air-velocity and deposition measurements	Airflow-field optimization, deposition prediction, and configuration assessment	[103,117,118,119,120]
Integrated autonomous airflow assistance	Autonomous hanging-track or unmanned-ground-platform sprayers with coordinated motion and airflow	System-level integration of platform motion, airflow, and spraying execution	[104,116]

**Table 7:** Classification of prescription-map-based coordinated control methods for variable-rate spraying.

Method Category	Representative Sensing/Control Strategies	Control-Oriented Outputs/Applications	Refs.
Remote-sensing vigor zoning and prescription generation	UAV multispectral/NDVI mapping combined with DOSAVIÑA or geostatistical zoning	Zone-specific application-rate prescriptions and field variable-rate implementation	[7,129,132,133]
Three-dimensional geometry/voxel prescription mapping	LiDAR or UAV 3D reconstruction with grid/voxel representations and spatial registration	Spatial dosage pre-allocation, spray-zone planning, and machine-readable prescriptions	[8,53,134]
Online sensor-based variable-rate execution	Ultrasonic sensing integrated with an online variable-rate sprayer	Real-time application-rate regulation during orchard operation	[135]
Multi-parameter coordinated prescription	Point-cloud analysis, leaf–wood separation, nozzle topology, airflow-demand estimation, and dosage control	Coordinated nozzle, liquid-flow, and airflow regulation within one prescription framework	[28]

**Table 8:** Classification of control-oriented perception-to-actuation pathways for coordinated orchard variable-rate spraying.

Main Regulated Variables	Control Application	Main Sensing Technologies	Core Descriptors/Perception Outputs	Information Class
Nozzle switching, PWM duty cycle, liquid flow rate, spraying region	Target detection, sectional control, canopy-volume-based variable rate, spray-zone/trajectory adjustment	LiDAR, ultrasonic sensing, RGB/RGB-D vision, UAV photogrammetry	Height, width, thickness, volume, boundary, spatial position; canopy profile/volume, target region and spray-zone boundary	Geometric profile
Liquid-flow allocation, air velocity/air volume flow rate/direction, nozzle combination	Layer-specific variable rate, nozzle-layout optimization, adaptive airflow-assisted regulation	3D LiDAR, RGB-D, ultrasonic sensing, point-cloud/vision fusion	Foliage density, depth, LAI/FAVD, porosity, permeability; layer/voxel density, leaf distribution and airflow-attenuation descriptors	Internal structure
Nozzle switching, dosage level, spray path, coordinated liquid–air parameters	Targeted spraying, priority-zone treatment, offline/online prescription-map control	RGB/deep learning, multispectral/hyperspectral sensing, UAV multimodal perception	Disease severity, leaf health, fruit/flower distribution, vigor/stress, treatment priority; target/risk maps and prescription maps	Status/prescription
Trigger timing, spray zone entry/exit, trajectory and spatial-offset compensation, safety constraints	Spatial registration, delay compensation, path/zone execution, platform-aware spraying	GNSS/RTK, INS, LiDAR-SLAM, radar, odometry, multimodal fusion	Tree/row position, platform pose/speed/attitude, terrain/obstacles, sensor–actuator offset; georeferenced tree maps, registration and timing correction	Spatial-geographic/operational context

**Table 9:** Cross-study quantitative synthesis of representative perception-to-actuation mappings and evidence strength.

Critical Interpretation	Reported Quantitative Outcome	Platform/Evidence Level	Perception → Actuation Link	Ref.
Clear perception-to-flow benefit; transferability beyond the tested vineyard configuration remains unproven.	57.4% reduction in plant-protection chemical consumption	Vineyard prototype/field-oriented test (B)	RGB-D canopy volume **→** PWM spray-flow regulation	[21]
Direct demand-to-dose linkage with agronomic spray-quality evidence; thresholds remain disease- and scenario-specific.	51.9% spray-volume reduction while maintaining spray quality	Pear air-assisted spraying/field test	Disease-severity classes from lesion recognition **→** PWM duty-cycle adjustment	[22]
Promising coupled liquid–air mapping; field robustness across cultivars and seasons remains to be demonstrated.	Approximately 42% pesticide saving with maintained deposition uniformity	Hardware-in-the-loop/structured-canopy evaluation	Leaf density, canopy depth and airflow attenuation **→** joint spray-volume and air-velocity prescription	[28]
Rare end-to-end sensing/control metrics; the deposition consequence of residual spatial/control error is not yet standardized.	2.84% volume-estimation error; 8.78% liquid-flow-rate control error; 160 ms response time	Orchard prototype	LiDAR canopy volume **→** PWM dosage with speed-adaptive delay compensation	[62]
Strong operational evidence for spatial prescription execution; not directly comparable with real-time canopy-sensor systems.	Field variable-rate implementation reported; outcome metrics are scenario-specific	On-farm vineyard field validation	UAV-derived vineyard vigor zones **→** prescription-map variable-rate application	[129]
Highlights the importance of spatial registration; standardized latency and deposition metrics are still needed.	Demonstrated georeferenced variable-rate execution	Orchard spatial–prescription/field-oriented system	UAV 3D/multispectral prescription + RTK registration **→** offline PWM variable-rate application	[53]

## Data Availability

The data presented in this study are available on request from the corresponding author. The data are not publicly available due to confidentiality and privacy restrictions.
